# Biologically Active Compounds from Probiotic Microorganisms and Plant Extracts Used as Biopreservatives

**DOI:** 10.3390/microorganisms11081896

**Published:** 2023-07-27

**Authors:** Desislava Teneva, Petko Denev

**Affiliations:** Institute of Organic Chemistry with Centre of Phytochemistry, Bulgarian Academy of Sciences, Laboratory of Biologically Active Substances, 139 Ruski Blvd., 4000 Plovdiv, Bulgaria; desislava.teneva@orgchm.bas.bg

**Keywords:** biopreservation, probiotic microorganisms, plant extracts, biologically active compounds

## Abstract

Ensuring the microbiological safety of food products is a pressing global concern. With the increasing resistance of microorganisms to chemical agents and the declining effectiveness of synthetic preservatives, there is a growing need for alternative sources of natural, bioactive compounds with antimicrobial activity. The incorporation of probiotics and plant extracts into food formulations not only enriches foodstuffs with microorganisms and phytochemicals with biologically active compounds, but also provides a means for product preservation. The current review considers the importance of the process of biological preservation for providing safe foods with high biological value, natural origin and composition, and prolonged shelf life, thereby improving consumers’ quality of life. To accomplish this goal, this review presents a series of examples showcasing natural preservatives, including beneficial bacteria, yeasts, and their metabolites, as well as phenolic compounds, terpenoids, and alkaloids from plant extracts. By summarizing numerous studies, identifying research challenges and regulatory barriers for their wider use, and outlining future directions for investigation, this article makes an original contribution to the field of biopreservation.

## 1. Introduction

The majority of commercially available food preservatives are chemically synthesized, and the food industry is currently seeking natural alternatives that do not have any detrimental effects on consumers or the environment [1]. Biopreservation involves the use of natural resources, such as plant and/or microbial constituents and/or their metabolites, to extend the shelf life and enhance the safety of food products [2]. Numerous microorganisms and plants have demonstrated antimicrobial properties [3]. By employing natural antimicrobial agents as food preservatives, it becomes possible to minimize excessive physical and chemical processing of products, thereby ensuring microbial safety. Traditional antimicrobials derived from natural resources, known for their pharmaceutical and biomedical activities, can be applied safely for food preservation, making them promising for both consumers and manufacturers. Biopreservatives can be broadly classified into two main categories based on their origin: those derived from microorganisms, and those derived from plants. The current review summarizes numerous studies and provides an overview of natural preservatives obtained from biologically active compounds found in probiotic microorganisms and plant extracts. It highlights their crucial role in the preservation of food products, identifying research challenges and regulatory barriers, and outlining future directions for investigation.

## 2. Biologically Active Compounds from Probiotic Microorganisms as Biopreservatives

Certain microorganisms have been found to produce various types of metabolites that possess antimicrobial properties. This has sparked growing interest in utilizing these antimicrobial substances to enhance the stability and safety of food products. For instance, compounds like lactic and acetic acids are extensively employed in numerous foodstuffs, while other antimicrobial substances are gaining significant attention as potential biopreservatives. These substances have the potential to serve as alternatives to nitrites, sulfites, parabens, and other conventional preservatives.

### 2.1. Bacterial Metabolites

Probiotic starter cultures not only contribute to the sensory quality of food but also serve as effective biopreservatives, enhancing the food’s shelf life. By introducing viable cells of mesophilic bacteria such as *Lactococcus lactis*, certain strains of *Lactobacillus*, and *Pediococcus* into refrigerated food storage (at temperatures below 5 °C), the growth of spoilage-causing bacteria and saprophytes can be significantly suppressed [4]. Additionally, the growth of pathogenic bacteria is reduced even at slightly higher temperatures (around 10–12 °C) [4]. Studies have demonstrated the beneficial effects of incorporating lactic acid bacteria into fresh meat, seafood, and certain processed meat products to combat pathogens such as *Clostridium botulinum, Salmonella serovars*, and *Staphylococcus aureus* [3]. Viable cells of *Lactobacillus, Lactococcus*, and *Leuconostoc* genera are added to fresh milk, meat, eggs, and seafood during refrigerated storage to inhibit the growth of psychrotrophic bacteria from the *Pseudomonas* genus [5]. This results in a 90% or greater reduction in the growth of psychrotrophic microorganisms during refrigerated storage for 4–10 days. The inhibitory activity can be attributed to the release of internal antimicrobial components, such as organic acids, bacteriocins, and hydrogen peroxide, from non-metabolizing lactic acid bacteria cells [5,6]. On the other hand, lactic acid bacteria are known to produce exopolysaccharides with varying compositions, structures, and molecular weights. While high-molecular-weight polysaccharides are commonly utilized in the food industry as texturizers, stabilizers, viscosifiers, and emulsifiers, their potential as preservative agents remains unexplored [5]. Although some studies have investigated the antimicrobial properties of exopolysaccharides, a definitive mechanism explaining their antimicrobial action against pathogenic microorganisms by lactic acid bacteria is yet to be established [5].

#### 2.1.1. Organic Acids, Diacetyl, Hydrogen Peroxide, and Reuterin

Organic acids: Lactic acid bacteria are well known for their ability to produce antimicrobial substances, including organic acids. For example, certain strains of the *Lactobacillus* genus secrete lactic acid, *Acetobacter aceti* produces acetic acid, and *Propionibacterium* sp. produce propionic acid. These microorganisms belong to the group of generally recognized as safe (GRAS) microorganisms and are commonly used as additives in many foods to enhance taste and extend shelf life, while also preventing the growth of undesirable microflora. These organic acids and their salts are typically added to foods at concentrations of about 1–2% [7]. Acetic acid and its salts find application in various food products. They inhibit the growth and viability of both Gram-positive and Gram-negative bacteria, as well as yeasts and molds. At a concentration of 0.2%, acetic acid exhibits bacteriostatic effects, while concentrations above 0.3% display bactericidal effects, especially at low pH values (pH < 4.5). Acetic acid is particularly effective against Gram-negative bacteria [4]. It can be used as an antimicrobial agent in dressings and mayonnaise. Propionic acid and its salts are primarily used as fungistatic agents, but they also inhibit the growth of pathogenic bacteria. At an acid concentration of 0.1–0.2% and pH 5.0, they exhibit increased sensitivity against Gram-negative bacteria. Propionic acid helps reduce the presence of molds and yeasts in dairy and cereal foods, non-alcoholic or low-alcohol beverages, and certain fresh fruits and vegetables [7]. Lactic acid, in addition to being used as a flavor enhancer in foods [4], possesses antibacterial properties even at concentrations of 1–2% and pH values above 5. It can inhibit the growth of both Gram-positive and Gram-negative bacteria, with a more pronounced bacteriostatic effect [7]. At lower pH values (pH < 4.5), lactic acid exhibits bactericidal activity, particularly against Gram-negative bacteria [7].

The antimicrobial effects of these organic acids are primarily attributed to their undissociated molecules. The dissociation constants (pKa) are 4.8 for acetic acid, 4.9 for propionic acid, and 3.8 for lactic acid [7]. In most foods with pH values of 5 and above, the undissociated fractions of these acids are relatively low, with lactic acid having the lowest. The lower antimicrobial activity of lactic acid is likely due to its low pKa [7]. The antimicrobial action of the undissociated molecules occurs when they enter the cytoplasm and dissociate, releasing hydrogen cations that disrupt the proton-motive force and internal pH, leading to protein denaturation and loss of bacterial viability [7]. These weak acids exhibit antimicrobial activity through the combined effects of undissociated molecules and dissociated ions, which can induce sublethal damage and increase the likelihood of bacterial loss of viability. Both undissociated molecules and dissociated ions contribute to cell damage [7]. Certain pathogenic bacterial species, such as *Salmonella typhimurium* and *Escherichia coli* O157:H7, possess resistance to low pH due to their ability to overproduce specific proteins induced by the acidic environment. These proteins, also known as stress proteins, enable these bacteria to survive at a lower internal pH [7].

Diacetyl: Diacetyl (2,3-butanedione), also known as *α*-diketone, is formed during the manufacture of various food products [5]. Diacetyl is produced in significant quantities by several species of lactic acid bacteria (*Lactococcus lactis* biovar. *diacetylactis*, *Lactobacillus paracasei*, *Lactobacillus bulgaricus*, Streptococcus thermophilus), especially during the metabolism of citric acid. It exhibits antibacterial activity against both Gram-positive and Gram-negative bacteria and is effective at concentrations around 0.1–0.25% [5]. Gram-negative bacteria are particularly sensitive to diacetyl at pH 5 or lower. When heated, its bactericidal capacity increases, but it is volatile and quickly loses effectiveness [5]. Under certain conditions, diacetyl can convert to acetoin (3-hydroxybutanone), which has reduced antibacterial effects [5]. The antibacterial action of diacetyl occurs through the deactivation of important enzymes. The dicarbonyl group (-CO-CO-) in diacetyl reacts with arginine in enzymes and modifies their catalytic centers [5].

Hydrogen peroxide: Some lactic acid bacteria (*Lactobacillus lactis*, *L. bulgaricus*, *Lactobacillus johnsonii*, *Lactobacillus acidophilus*) produce hydrogen peroxide under anaerobic growth conditions [5]. Due to the lack of cellular catalase, pseudocatalase, or peroxidase, cells of lactic acid bacteria release hydrogen peroxide into the medium. Hydrogen peroxide possesses strong oxidizing properties and exhibits antimicrobial activity against bacteria, molds, and viruses, including bacteriophages [4]. Its antimicrobial action is attributed to its powerful oxidizing ability, which disrupts cellular components [4]. Certain strains can produce enough hydrogen peroxide to induce bacteriostatic effects (6–8 µg/cm^3^), but bactericidal effects (30–40 µg/cm^3^) are rarely achieved [4]. Under aerobic conditions, hydrogen peroxide is produced in minimal amounts. It is permitted for use as an antimicrobial agent in the refrigerated storage of raw milk and eggs (at concentrations of about 25 ppm). Prior to pasteurization, catalase (0.1–0.5 g/455 kg) is added to remove any residual peroxide. However, its use in food preservation is limited [4].

Reuterin: *Lactobacillus reuteri*, which can be of human or animal origin, produces a small molecule called reuterin (beta-hydroxypropionaldehyde; CHO–CH_2_–CH_2_OH), which exhibits antimicrobial effects against both Gram-positive and Gram-negative bacteria. Reuterin’s antimicrobial action stems from its ability to inactivate essential enzymes such as ribonucleotide reductase. Reuterin is only produced when glycerol is present in the growth medium, which limits its use in food preservation. However, studies have shown that foods supplemented with glycerol and inoculated with reuterin-producing *L. reuteri* can effectively control the growth of undesirable bacteria [4].

#### 2.1.2. Bacteriocins from Lactic Acid Bacteria

Bacteriocins are bioactive peptides produced by various bacterial species, including lactic acid bacteria and some propionic acid bacteria. These bacteriocins have attracted significant interest in microbiology due to their inhibitory effects on various Gram-positive and Gram-negative pathogenic bacteria found in foods product [8]. Many bacteriocin-producing strains belonging to different genera and species of lactic acid bacteria have been isolated, including *L. lactis*, *S. thermophilus*, *L. acidophilus*, *Lactobacillus plantarum*, *Lactobacillus sake*, *Lactobacillus curvatus*, *Leuconostoc mesenteroides*, *Leuconostoc carnosum*, *Leuconostoc gelidum*, *Pediococcus acidilactici*, *Pediococcus pentosaceus*, *Pediococcus parvulus*, *Enterococcus faecalis*, *Enterococcus faecium*, and *Bifidobacterium bifidum* [9]. Bacteriocins are synthesized in ribosomes and are positively charged particles. They can have *α*-helix, *β*-sheet, or both structures and may contain thioether linkages, disulfide bridges, or free thiol groups. The presence of an α-helical structure with opposite polar and nonpolar sides along the long chain enables bacteriocins to interact with both aqueous and lipid phases [10]. The complete amino acid sequences of many bacteriocins are not yet fully elucidated. However, approximately 45 bacteriocins have known sequences, including nisin, lactacin, pediocin, leucocin, sakacin, lactococcin A, bifidocin B, carnobacteriocin B2, bavaricin, and plantaricin. 

**Figure 1:**
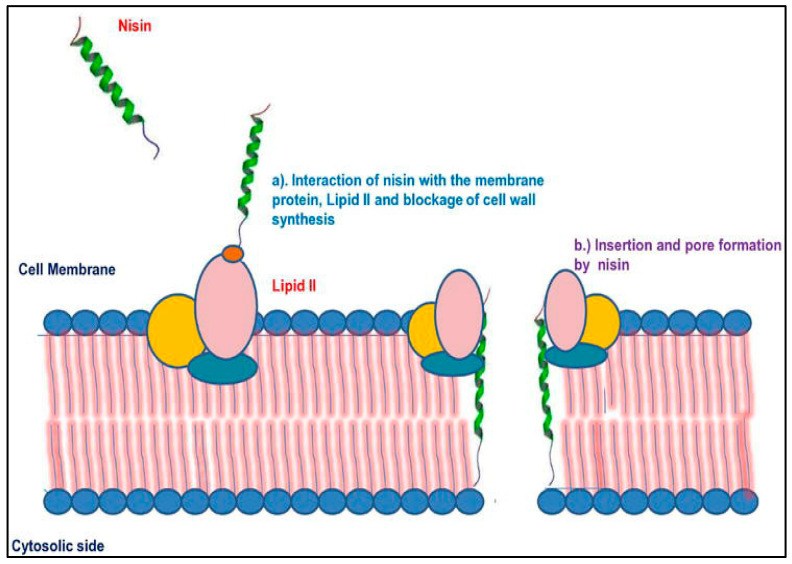
Mechanism of action of class I bacteriocins (nisin) according to Sidhu and Nehra [10].

The bactericidal activity of bacteriocins is not necessarily correlated with the number of amino acids in their structure [10,11]. Bacteriocins can be classified into four main classes based on their properties, post-translational modifications, and the number of peptides in their mature molecule: Class I: lantibiotics; Class II: small, heat-stable peptides; Class III: high-molecular-weight, heat-labile peptides; Class IV: complex bacteriocins, including non-protein components such as lipids or carbohydrates [11,12]. Bacteriocins produced by lactic acid bacteria exhibit strong antimicrobial activity against various pathogens. Gram-positive bacteria are generally more sensitive to bacteriocins, while Gram-negative bacteria are usually resistant [10]. However, Gram-negative bacteria can become sensitive to bacteriocins under physical or chemical stress, which alters the structure of their cell-surface lipopolysaccharides. The bactericidal effect against susceptible bacterial cells is achieved by destabilizing the cytoplasmic membrane [10]. Initially, bacteriocin molecules adsorb to the membrane surface and form temporary pores, resulting in the loss of the proton-motive force and pH gradient across the membrane [10]. This leads to membrane permeability changes, nutrient deprivation, altered ATP synthase function and, ultimately, lysis and death of the susceptible bacterial cell. The exact mechanisms of these changes may vary depending on the class of bacteriocin [10]. For example, the mechanism of action of the class I bacteriocin nisin involves initial binding to lipid II in the cell wall, allowing the molecules to make contact with the membrane and form pores (Figure 1) [10]. Nisin is more effective against bacteria in the stationary phase of growth, while some bacteriocins, such as pediocin, can act independently of the membrane potential difference, affecting both the stationary and exponential growth phases [10].

Some bacteriocins demonstrate bactericidal effects towards related species and strains [13,14,15]. There are species that produce more than one bacteriocin (e.g., *L. lactis* produces lactococcins A, B, and M), and strains of the same species can synthesize different bacteriocins (sakacin A and sakacin P are produced by different strains of *L. sake*) [9]. The efficacy of a bacteriocin preparation containing nisin and pediocin was tested against pathogenic bacteria in processed meat products during refrigerated storage. The results demonstrated a reduction in the concentrations of Gram-positive bacteria, including *Lactobacillus mesenteroides* and *Listeria monocytogenes*, as well as Gram-negative pathogens such as *Salmonella* sp. and *E. coli*, over a six-week storage period at 4 °C [16]. Nisin has also been found to suppress *L. monocytogenes* in ready-to-eat chicken meat and control the growth of *Alicyclobacillus acidoterrestris* in orange juice [17]. Encapsulated nisin combined with low temperatures has been applied to combat pathogenic *L. monocytogenes* in milk [18]. The antimicrobial activity of nisin against *Salmonella enteritidis* in ground sheep meat was enhanced when combined with oregano essential oil [19]. In another study, the application of pediocin AcH, produced by *Lactobacillus plantarum* WHE92, inhibited *L. monocytogenes* in fresh cheese [20]. Comparisons have been made between lacticin, nisin, and the preservative sodium metabisulfite, demonstrating the effectiveness of lacticin and nisin against Gram-positive bacteria in pork [21,22]. Combining organic acids with any of these bacteriocins has been shown to increase their antimicrobial activity against *Listeria innocua, Salmonella kentucky,* and *Clostridium perfringens* [21,22].

### 2.2. Yeast Metabolites

Some yeast species, including strains of *Saccharomyces cerevisiae*, produce proteins with antimicrobial properties (known as killer toxins or zymocins), as well as inhibiting pathogenic enzymes. *P. pentosaceus* and *S. cerevisiae* strains in sourdough bread inhibited the growth of *Aspergillus flavus* by 56.4% in 96 h. Yeast cells were found to adhere tightly to the mold mycelium and produce *β*-glucanase, which destroys the cell wall of the mold [23]. Younis et al. [24] analyzed the antimicrobial activity of yeasts isolated from different food groups against pathogenic bacteria. The results showed high activity against *S. aureus* and *E. coli*, and weaker activity against *Pseudomonas aeruginosa*. Ng et al. [25] investigated the antimicrobial and antioxidant activities of phenolic metabolites secreted from a naringenin-producing *S. cerevisiae* strain (a GRAS organism) against the pure flavonoid naringenin and its prenylated derivatives in vitro, to assess their potential as natural food preservatives. The yeast *Pichia anomala* has been isolated from different environments, e.g., plants, fruits, human intestinal tracts, and dairy products. Its antagonistic activities against many spoilage organisms of plant products for food or feed are exhibited by producing ethyl acetate, ethanol, and carbon dioxide [26]. Some species of yeast produce different colored pigments, such as astaxanthin (a red pigment extracted from the yeast *Phaffia* sp.). Astaxanthin is used as a natural colorant in food products [27]. Another red pigment—monask—is produced by the yeast *Monascus* sp. It is used in China for the preparation of red rice wine [28].

## 3. Biologically Active Compounds of Plant Origin Used as Biopreservatives

The study of the natural antimicrobial properties of biologically active compounds of plant origin and their use as biological preservatives is of increasing interest for the food industry. Medicinal plants produce a wide range of antibacterial, antifungal, and antiviral compounds. The majority of essential oils and plant extracts obtained from non-toxic herbs and spices have GRAS status. Their addition to food products as preservative agents is based on their aroma–flavor effect. The effectiveness of biopreservatives of plant origin depends on various factors, such as concentration, mode of action, storage, and application methods. Most biologically active compounds are produced as secondary metabolites, giving plants their color, taste, and aroma. They are thoroughly studied for their potential as additives in the preparation of foods, modifying their sensory profiles and increasing their durability. The antimicrobial and antioxidant properties of essential oils and extracts are due to substances of different chemical composition, e.g., phenolic compounds, terpenoids, and alkaloids [29,30].

### 3.1. Polyphenolic Compounds

Polyphenols are a large group of secondary metabolites that are produced by plants in response to various environmental factors (light, cold, pollution, etc.). They contain more than one phenolic hydroxyl group attached to one or more benzene rings. In terms of structure and reactivity, polyphenols include polyhydroxy aromatic compounds with several phenolic nuclei. They protect chloroplasts from photodegradation by absorbing high-energy irradiation, and by reacting with free radicals and reactive oxygen species [31]. Phenolic compounds provide protection against the harmful effects of UV light and against pathogenic insect attacks, and they are known antimicrobial metabolites. They exhibit low toxicity to higher organisms and strong antimicrobial activity against pathogenic microorganisms such as *Salmonella enteritidis*, *Listeria monocytogenes*, *Pseudomonas fluorescens*, *Aeromonas hydrophila*, *Bacillus cereus*, *Escherichia coli*, and *Enterobacter aerogenes* [32,33,34]. They affect the permeability of the cell membranes of microorganisms and inhibit the generation of ATP by disrupting the proton-motive force [32]. In addition, plant phenolics can have specific benefits for human health. Therefore, they can be used not only as antimicrobial and antioxidant agents, but also as antitumor, antihypertensive, antimutagenic, and other agents [33,34]. 

**Figure 2:**
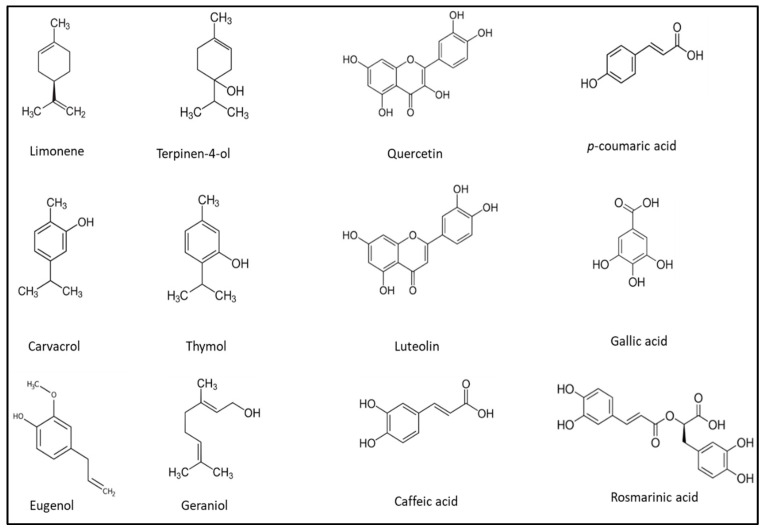
Structural formula of some of the most common phytochemicals in essential oils and plants extract, acting as biopreservatives.

Various phenolic compounds are found in essential oils and plant extracts, including quercetin, gallic acid, rosmarinic acid, cinnamic acid, thymol, citronellin, citral, lupine, luteolin, vanillin, benzoic acid, coumarins, eugenol, etc. The structural formula of some of the most common molecules in essential oils and plant extracts are presented in Figure 2.

The numerous studies conducted with plant extracts and essential oils containing various biologically active substances have shown effective results in controlling the colonial growth of pathogenic microorganisms. For example, rosmarinic acid-containing extracts have been widely investigated as natural antioxidants and biopreservatives in food products [35]. Similarly, eugenol has been found to play an important role in the inhibition of *L. monocytogenes* in meat [36]. Some essential oils inhibit the growth of the pathogens *S. enteritidis*, *L. monocytogenes*, *P. fluorescens*, *A. hydrophila*, *B. cereus*, *E. coli*, and *E. aerogenes* in various foods [34,37]. Gram-positive bacteria are sensitive to the tested extracts and oils, while Gram-negative bacteria are less affected. This is due to the differences in the structure and composition of the cell walls of the two groups of bacteria. The presence of an outer membrane in Gram-negative bacteria impairs the diffusion through the membrane to the cytoplasm of the cell, which makes them more resistant to the action of the extracts and oils studied [34,37]. 

Flavonoids and their derivatives are the richest group of plant pigments, ranging in color from pale yellow (isoflavones) to deep yellow (flavones, flavonols, chalcones, aurones), to orange (aurones), to red and blue (anthocyanins). Flavones consist of two benzene rings (A and B) linked by a *γ*-pyrone C-ring, and the different subgroups of flavonoids are classified according to the substitution patterns in the C-ring. Both the oxidation of the heterocyclic ring and the positions of the B-ring groups are important for classification. Chalcones, isoflavones, and aurones belong to the same chemical group. They do not possess the 2-phenyl-benzopyrone backbone, but they are closely related to flavonoids, both chemically and biosynthetically. Recent studies have included flavonoids in the group of biopreservatives. Tosi et al. [38] analyzed a phenolic/flavonoid extract obtained from propolis that contained a total of 32.31% soluble compounds (2.1% coumaric and syringic acids, 5.16% quercetin, 0.47% apigenin, 8.15% galangin, 7.2% caffeic acid and chrysin, and 9.23% other mixed phenolic compounds). The correlation between the inhibition of bacterial growth and the concentration of the extract showed that this extract is a successful antimicrobial agent against *E. coli* and, therefore, can be used as a natural preservative in food products. 

The presence of mycotoxins in food can seriously endanger human health, causing various adverse effects, such as mutagenicity, as well as oncological, gastrointestinal, and kidney diseases. El-Nagerabi et al. [39] reported that extracts of roselle (*Hibiscus sabdariffa* L.) and black cumin (*Nigella sativa* L.) essential oil affected the in vitro growth of *A. flavus* and *Aspergillus parasiticus* and the production of aflatoxin B1. *Peltophorum ferrugineum* (Decne.) Benth. flower extracts exhibited in vitro antibacterial activity against strains of *B. cereus*, *S. aureus*, *E. coli*, and *Yersinia enterocolitica*, as well as an antimutagenic effect against *S. typhimurium* [40]. Polyphenolic compounds found in common essential oils such as basil (*Ocimum basilicum* L.), dill (*Anethum graveolens* L.), tangerine (*Citrus reticulata* L.), grapefruit (*Citrus paradise* L.), lemon (*Citrus lemon* L.), and cinnamon (*Cinnamomum zeylanicum* Blume) demonstrated in vitro antimicrobial activity against pathogenic bacteria and saprophytic microorganisms, including spore-forming bacteria, molds, and yeasts [41,42,43]. *Borago officinalis* L., applied as a freshly squeezed juice, has also been recognized for its potential as a biopreservative in food production. Micelli et al. [44] examined the inhibitory activity of an aqueous extract of *Borago officinalis* L. against foodborne pathogens in vitro and reported strong preservative effects. Similarly, vanillin and geraniol demonstrated in vitro antimicrobial properties against *E. coli* O157:H7 in strawberry juice [45]. The biochemical and molecular characterization of extracts from *Brassica oleracea* L. and *Beta vulgaris* L. was investigated by Sarhan et al. [46], with the aim of exploring their potential as biological food preservatives as natural alternatives to synthetic preservatives and colorings. Polyphenol-rich essential oils and extracts derived from *Matricaria recutita* L. and *Foeniculum vulgare* Mill. have exhibited in vitro antioxidant and preservative properties in yogurt samples [47]. Additionally, *Mentha piperita* L. essential oil has shown efficacy as a preservative agent for ground beef during storage, either alone or in combination with bacteriocins [48]. These studies highlight the diverse range of natural compounds and extracts that can serve as effective alternatives to synthetic preservatives in various food applications.

### 3.2. Terpenes

Terpenes are the most diverse class of biologically active compounds. They are classified according to the number of carbon atoms and according to the presence of isoprene residues: (1) monoterpenes contain 10 carbon atoms or two isoprene units; (2) sesquiterpenes contain 15 carbon atoms or three isoprene residues; (3) diterpenes contain 20 carbon atoms or four isoprene residues; (4) triterpenes contain 30 carbon atoms or six isoprene units; (5) tetraterpenes contain 40 carbon atoms or eight isoprene units (Table 1). Although terpenes are secondary metabolites, they play a major role in plant growth [49,50]. Gibberellins (diterpenes) are plant hormones, and sterols (triterpene derivatives) are responsible for cell stabilization, while carotenoids (tetraterpenes) exhibit a protective function against photo-oxidation [50,51]. Carotenoids, such as *β*-carotene, lutein, lycopene, or zeaxanthin, are fat-soluble pigments found in vegetables and fruits, giving them yellow, orange, and even red colors. On the other hand, some terpenes are toxic and exhibit a protective function against pathogenic microorganisms. The most important of all terpenes are the volatile monoterpenes and sesquiterpenes included in the composition of essential oils. Monoterpenes and sesquiterpenes are well-known bioactive compounds and are used as biopreservatives in the food industry [51].

Our recent research findings provide insights into the chemical composition and antimicrobial activity of *Citrus aurantium* L. essential oil and the monoterpene limonene, via the disc diffusion method. We observed that *Citrus aurantium* L. oil exhibited higher in vitro antimicrobial activity compared to limonene, which we attributed to the synergistic interaction between individual terpenes and the influence of non-volatile components present in the oil [58]. Additionally, we conducted studies to determine the chemical composition of basil (*Ocimum basilicum* L.) and dill (*Anethum graveolens* L.) oils, assessing their potential as preservatives in mayonnaise. In *Ocimum basilicum* L. essential oil, we identified 20 compounds, with methyl chavicol (36.81%), methyl eugenol (20.40%), *β*-linalool (14.35%), and eugenol (10.55%) dominating among the monoterpene hydrocarbons. *Anethum graveolens* L. oil contained 17 components, with carvone (39.05%), limonene (21.11%), and *γ*-phellandrene (22.68%) as the predominant monoterpene hydrocarbons [41]. In our investigations of rosemary (*Rosmarinus officinalis* L.) essential oils, we identified numerous compounds, with *ρ*-cymene being the most prevalent component, constituting 42.95% (leaf) and 42.35% (flower). *ρ*-Cymene exhibits various biological activities, including antioxidant and antimicrobial properties [42]. Singh et al. [59] described the in vitro antifungal and toxigenic activity of oils derived from *Citrus maxima* Burm. *and Citrus sinensis* L. Osbeck, as well as their main component, *α*-limonene. These essential oils were found to completely inhibit the production of aflatoxin B1 at concentrations of 500 μL/L, while *α*-limonene achieved the same effect at lower concentrations (250 μL/L) [59]. The fungicidal and bactericidal activities displayed by monoterpenes make them potential candidates for use as biopreservatives in food emulsions.

Saponins, a group of compounds found in plants, possess insecticidal, antiviral, antibacterial, and antifungal activities. They consist of a triterpene or steroidal aglycone and a sugar chain primarily composed of rhamnose, xylose, arabinose, galactose, and fucose. Their amphiphilic nature arises from the presence of a hydrophobic aglycone and a hydrophilic carbohydrate chain, making them suitable as emulsifiers, foaming agents, and detergents [60]. In recent years, there has been increasing interest in the use of saponins as natural emulsifiers, flavoring agents, and preservatives in food and beverage products. Plant species such as *Quillaja saponaria* and *Yucca schidigera*, which produce saponins, have attained GRAS status and are permitted for use in the food industry in the USA. Saponins isolated from yucca (*Yucca elephantipes*) have been shown to inhibit food oxidation caused by yeasts such as *Debaryomyces hansenii, Pichia nakazawae, Zygosaccharomyces rouxii*, *Candida famata,* and *Hansenula anomala* [61,62,63]. In Japan, *Yucca schidigera* extract is employed as an antimicrobial agent against molds and yeasts present in cooked rice, pickled vegetables, and fish. Treatment of yeast with *Quillaja saponaria* extract increases the permeability of cell membranes, facilitating the production of yeast lysates [61,62,63].

### 3.3. Alkaloids

Alkaloids are a diverse group of biologically active natural compounds that possess a wide range of chemical structures. These compounds are considered to be secondary metabolites that are derived from various amino acids. Alkaloids are classified based on the amino acid from which they originate and the position of the nitrogen atom within their molecular structure [64,65,66]. Commonly, alkaloids are derivatives of amino acids such as lysine, ornithine, tyrosine, tryptophan, histidine, phenylalanine, nicotinic acid, and anthranilic acid. Furthermore, alkaloids can be categorized into different groups based on their biosynthesis. For instance, indole alkaloids, such as ergometrine, are derived from tryptophan, while piperidine alkaloids like lobeline originate from lysine. Pyrrolidine alkaloids, such as hygrine, are derived from ornithine, and phenylethylamine alkaloids are derived from tyrosine. Additionally, imidazole alkaloids, like pilocarpine, are derived from thydine. It is important to note that structurally similar alkaloids may have distinct biosynthetic pathways and exhibit different biological activities. Alkaloids are believed to play a role in regulating the growth of plant species and have a wide range of activities, such as hypolipidemic, gastrointestinal regulation, anti-inflammatory, and antimicrobial functions [64,65]. 

*Rhizoma coptidis* (derived from the roots of *Coptis chinensis*, *Coptis deltoidea*, or *Coptis teeta*) extract is commonly utilized to improve blood circulation, eliminate blood stasis, and provide nourishment to the kidneys and intestines. The primary active components found in *Rhizoma coptidis* are alkaloids, such as berberine, coptisine, palmatine, and epiberberine [64]. These compounds have demonstrated significant hypolipidemic effects, effectively lowering lipid levels. Additionally, *Rhizoma coptidis* alkaloids possess strong gastrointestinal regulatory properties. When administered to hyperlipidemic mice fed a high-fat, high-cholesterol diet, the alkaloids extracted from *Rhizoma coptidis* effectively reduced lipid levels by modulating bile acid pathways [64,65]. A study investigating the antimicrobial activity of caffeine extracted from green tea (*Camellia sinensis* L.) and coffee (*Coffea arabica* L.) extracts against pathogenic microorganisms, including *E. coli*, *Proteus mirabilis*, *Klebsiella pneumonia*, and *Pseudomonas aeruginosa*, revealed a bactericidal effect. The minimum inhibitory concentration (MIC) values ranged from 65.5 to 250 μg/mL for caffeine isolated from coffee, and from 65.5 to 500 μg/mL for caffeine derived from green tea [66]. 

## 4. Practical Examples for the Application of Probiotic Microorganisms and Plant Extracts Used as Biopreservatives

### 4.1. Application of Microorganisms

The utilization of plant extracts, essential oils, and beneficial microorganisms that produce various metabolites has ushered in a new era of functional additives and biological preservatives. Extensive literature exists on the practical application of microbial and plant metabolites and extracts for the biopreservation of food products. Table 2 provides an overview of the application of commonly used probiotics and beneficial microorganisms in food products. Scientific research has convincingly demonstrated the potential health advantages of incorporating probiotics into various food matrices. However, it is crucial to highlight that the application of probiotics for disease prevention and treatment should always be guided by medical professionals and subsequently endorsed by the food industry.

The food industry has shown significant interest in the potential use of bacteriocins produced by probiotics as natural preservatives [8,67]. Probiotic bacteria are incorporated into a wide range of functional foods, including yogurts, cheeses, and mousses, as well as non-dairy products such as cereals, fruit and vegetable juices, chocolates, mayonnaise, and meat products [41,68,69,70,71,72,73,74,75,76,77]. While probiotics have traditionally been delivered through fermented and non-fermented foods, the modern food industry aims to integrate probiotic microorganisms into non-fermented food products. Additionally, probiotics are employed in the form of functional foods and pharmaceuticals [78].

**Table 2:** Example of the use of probiotics for biopreservation of different foodstuffs.

Food Substrates	Probiotics Used in Biopreservation	Observed Biopreservation Effect	References
Mayonnaise	*L. plantarum*	Exclusion of mesophilic aerobic and facultative anaerobic microorganisms	[41,68]
	*L. casei* and *L. acidophilus*	Exclusion of pathogenic microorganisms	
Fresh pork sausages	*L. sakei*	Exclusion of pathogenic microorganisms	[69,70]
Fermented sausage	*L. plantarum*		
Yoghurt	*Propionibacterium jensenii*	Exclusion of spoilage molds and yeasts	[71]
Cheese	*L. paracasei* subsp. *paracasei*		
Bread	*L. plantarum*,	Exclusion of spoilage molds and yeasts	[72]
	*L. paracasei* and		
	*Leuconostoc mesenteroides*		
Chocolates	*L. delbrueckii* subsp. *bulgaricus*	Fermentation—low pH Exclusion of mesophilic aerobic and facultative anaerobic microorganisms	[73,74]
Chocolate mousses	*L. plantarum*		
Cantaloupe juice	*L. casei*	Fermentation—low pH	[75,76,77]
Orange juice	*P. acidilactici*		
Pineapple juice	*L. casei*		

### 4.2. Application of Plant Extracts

Plant extracts and essential oils are commonly incorporated into the preparation of food products to enhance their characteristics. Furthermore, the antioxidant properties exhibited by the molecules present in these plant-based ingredients can play a significant role in preventing natural products’ deterioration, especially lipid oxidation, benefiting the end user [79]. Throughout history, medicinal plants and spices have been employed to manage microbial changes and harness their antimicrobial activity in food preservation. Plant extracts and oils derived from basil (*Ocimum basilicum* L.), dill (*Anethum graveolens* L.), grapefruit (*Citrus paradise* L.), lemon (*Citrus lemon* L.), yarrow (*Achillea millefolium* L.), garlic (*Allium sativum* L.), onion (*Allium cepa* L.), eucalyptus (*Eucalyptus globulus* Labill.), juniper (*Juniperus communis* L.), mint (*Mentha piperita* L.), rosemary (*Rosmarinus officinalis* L.), sage (*Salvia officinalis* L.), clove (*Syzygium aromaticum* L.), thyme (*Thymus vulgaris* L.), lavender (*Lavandula angustifolia* L.), and lemon balm (*Melissa officinalis* L.) have demonstrated the ability to extend the shelf life of various food products. These extracts and oils exhibit bacteriostatic and fungistatic effects, attributed to the presence of biologically active compounds such as phenols, aldehydes, ketones, and terpenes [41,74,79,80,81,82,83,84,85,86,87,88]. The application of these natural extracts has been explored for the preservation of cheeses, mousses, cereals, fruit and vegetable juices, mayonnaise, and meat products (see Table 3). In addition to effectively controlling undesirable microflora, plant extracts and oils play a crucial role in enhancing product quality and prolonging shelf life. Moreover, these extracts and oils not only possess antimicrobial properties against specific food pathogens but also have a stimulating effect on lactic acid bacteria, which are widely utilized as biopreservatives in various food applications [79].

### 4.3. Combined Application of Probiotic Microorganisms and Plant Extracts

The combined application of probiotic microorganisms and plant extracts synergistically enhances the antimicrobial properties of biopreservatives. In our recent study, we demonstrated the biopreservative effects of the probiotic *L. plantarum* LBRZ12 and basil (*Ocimum basilicum* L.) and dill (*Anethum graveolens* L.) essential oils during refrigerated storage of mayonnaise for 40 days [41]. In terms of pathogenic microorganisms, molds, and yeasts, the mayonnaise met the standard requirements, but the number of live cells of mesophilic aerobic and facultative anaerobic microorganisms was high (10^4^ cfu/g). It was observed that the unwanted microflora in mayonnaise preserved with lactobacilli and essential oils gradually decreased during the storage period and completely disappeared after the 20th day. This could be explained by the antimicrobial activity of the probiotic strain *L. plantarum* LBRZ12 against mesophilic aerobic and facultative anaerobic microorganisms, as well as the beneficial effects of essential oils. Another study showed that the probiotic strain *L. plantarum* D2, added alone or together with essential oils of grapefruit (*Citrus paradise* L.) and lemon (*Citrus lemon* L.), provides biological preservation of chocolate mousses, maintaining a high concentration of viable cells (10^5^–10^6^ cfu/g) in the process of storage in refrigerated conditions for 20 days [74]. Kuley et al. [86] investigated the effects of *L. plantarum* FI8595 and *P. acidilactici* ATCC 25741, alone or in combination with *Thymus vulgaris L.* and *Laurus nobilis* L. extracts, on the food safety and the sensory, microbiological, and chemical quality of fermented and vacuum-packaged sardine fillets. The results revealed that the use of *L. plantarum* and *P. acidilactici* in combination with thyme extract was the most effective way to improve the shelf life of fermented fish [86]. 

Herbal extracts and essential oils not only enhance the sensory and nutritional properties of food products but also improve the viability and stability of probiotic microorganisms incorporated into them. A study by Chaikham et al. [87] explored the effects of Thai herbal extracts (cashew flower (*Anacardium occidentale* L.), yanang (*Tiliacora triandra*), pennywort (*Centella asiatica* (Linn.) Urban), and green tea (*Camellia sinensis* L.) on the survival of probiotic bacteria (*Lactobacillus casei* 01, *L. acidophilus* LA5, and *Bifidobacterium lactis* Bb-12) added to fruit juices and mixed sour milks during refrigerated storage. It was observed that yogurt supplemented with 0.05% (*w/v*) cashew blossom extract exhibited a higher number of viable *L. casei* cells, while green tea extract improved the stability of probiotic microorganisms in yogurt. Another study evaluated the impact of aqueous extracts of *Allium sativum* L. and *Cinnamomum verum* L. on the survival of the probiotic strain *Bifidobacterium bifidum* in cow and camel milk yogurts during refrigerated storage for 21 days [88]. The presence of herbal extracts positively influenced the viability of probiotic cells in the fermented milk products. The addition of *Allium sativum* L. and *Cinnamomum verum* L. extracts did not affect the number of viable *B. bifidum* cells, indicating that the bioactive compounds present in these extracts may contribute to reducing oxidative stress in the yogurt environment, thereby preserving the probiotic cells [88].

## 5. Regulatory Status of Microbial and Plant Biopreservatives

From the conducted review of the literature, it is evident that many microbial strains and plant extracts have been investigated for their potential to act as biopreservatives. However, it should be noted that microbial and plant-based biopreservatives are considered to be food additives, and their practical application in the food industry is subject to regulation by various authorities worldwide, including the Food and Drug Administration (FDA) in the United States and the European Food Safety Authority (EFSA). It is important to note that the regulatory status of microbial and plant-based biopreservatives may vary between countries and regions, and manufacturers must comply with the applicable regulations in each market where their products are sold. Achieving the right balance between effective dosage and potential toxicity is crucial when incorporating these substances into food products. 

In 2002, Mogensen et al. compiled a list of microorganisms and their metabolites, such as organic acids (e.g., lactic acid, acetic acid, formic acid, propionic acid), bacteriocins, etc., that have a documented history of safe use as biopreservatives [89,90]. The FDA has granted generally recognized as safe (GRAS) status to nisin and lacticin, deeming them harmless antimicrobial agents [91,92,93]. Currently, in the EU, the following microbial metabolites are used as food preservatives: nisin (E234), natamycin (E235), and propionic acid (E280), whereas acetic acid (E260) and lactic acid (E270) are approved as acidity regulators. 

The FDA has also documented various essential oils and plant extract compounds as natural biopreservatives in food products [94]. Aromatic compounds such as pinene, limonene, linalool, thymol, carvacrol, eugenol, vanillin, citral, cinnamaldehyde, menthol, and lavenderol have been reported to pose no risk to human health. The European Commission also recognizes these compounds as GRAS and regularly updates a list of approved substances [95]. Basil, rosemary, oregano, clove, lavender, and cinnamon are among the plant extracts and essential oils recognized as GRAS [94]. Regardless of the huge volume of scientific information about the effectiveness of plant extracts as biopreservatives, in the EU, only one plant extract is officially recognized as a food additive. This is rosemary (*Rosmarinus officinalis* L.) extract (E392), used as a preservative and antioxidant in foodstuffs. It should be noted that most studies conducted with many of these compounds, especially plant extracts, have used in vitro tests that differ significantly from manufactured or prefabricated products. It is important to note that in vitro studies alone are insufficient to confirm the antimicrobial activity of products, and it is not an easy task to extrapolate the results of these tests to their actual application in food products. The biggest regulatory barriers to the wider use of plant extracts in the food industry as food preservatives are probably related to their standardization and the right balance between effective dosage/concentration and toxicity.

## 6. Conclusions

Biopreservatives incorporating probiotic microorganisms and plant extracts have emerged as effective strategies to extend the shelf life of foodstuffs. These natural compounds possess antimicrobial properties that can inhibit the growth of spoilage and pathogenic microorganisms. Probiotic microorganisms such as lactobacilli, bifidobacteria, and yeasts are capable of producing organic acids, diacetyl, hydrogen peroxide, reuterin, bacteriocins, etc., with bacteriostatic, bactericidal, fungistatic, and fungicidal effects, making them valuable natural biopreservatives for application in the food industry. Plant extracts, including essential oils, as well as isolated biologically active compounds derived from aromatic and medicinal plants, also demonstrate significant antimicrobial properties, making them effective biopreservatives. Polyphenolic compounds, terpenoids, and alkaloids present in plant extracts offer both antioxidant and antimicrobial properties, further enhancing their ability to combat pathogenic and spoilage microorganisms. Consequently, they can serve as natural preservatives when used in small quantities, thereby contributing to enhanced food safety. The combination of probiotic microorganisms and plant extracts is an interesting strategy to synergistically enhance the antimicrobial properties of biopreservatives. By utilizing these biologically active compounds, the reliance on chemical preservatives can be reduced, thereby improving the safety and overall quality of food products. However, there is a huge gap between the research on biopreservatives and their practical application in the food industry, which is regulated by legislation, especially in the EU. The biggest regulatory barriers to the wider use of plant extracts in the food industry as food preservatives are probably related to their standardization and the right balance between effective dosage and toxicity. Therefore, it is crucial to conduct further research to optimize the application of these compounds in food products and evaluate their long-term effects on human health. This research will enable us to fully harness the potential of biopreservatives, ensuring their safe and effective utilization in the food industry.

## Figures and Tables

**Table 1:** Different types of terpenes and their properties.

Classification	Carbon Atoms	Identification	Application and Uses	References
Monoterpenes	C_10_	Essential oils and plant extracts	Flavorings and antimicrobial agents	[52,53]
Sesquiterpenes	C_15_	Essential oils and plant extracts	Antimicrobial agents	[54]
Diterpenes	C_20_	Bitter plant substances	Anti-inflammatory and antimicrobial agents	[55,56,57]
Triterpenes	C_30_	Essential oils and plant extracts	Emulsifiers, flavorings, and preservatives	[50,51]

**Table 3:** Essential oils and plant extracts used in biopreservation of different food substrates.

Food Substrates	Essential Oil/ Plant Extract Used for Biopreservation	Observed Biopreservation Effect	References
Mayonnaise	Dill and basil oils	Exclusion of mesophilic aerobic and facultative anaerobic microorganisms Inhibition of lipid oxidation	[41,80]
	Yarrow oil		
Sausages	Garlic and onion plant extracts	Exclusion of pathogenic microorganisms	[79,81,82]
Minced beef meat	Eucalyptus, juniper, mint, rosemary, sage, clove, and thyme oils		
Pork fillets	Oregano oil		
Cheese	Eucalyptus, juniper, mint, rosemary, sage, clove, and thyme oils	Exclusion of pathogenic microorganisms	[81]
Bread	Lavender and melissa plant extracts	Exclusion of spoilage molds and yeasts	[83]
Chocolate mousses	Lemon and grapefruit oils	Exclusion of mesophilic aerobic and facultative anaerobic microorganisms	[74]
Watermelon juice	Melissa oil	Exclusion of pathogenic microorganisms	[84,85]
Pineapple and mango juice	Wild mint and peppermint oils		

## Data Availability

Data sharing not applicable.
